# Supporting the early development of decentralised oncology units in Portuguese-speaking African countries: initial results of the GONCO program in Angola and Cape Verde

**DOI:** 10.3332/ecancer.2050

**Published:** 2025-11-28

**Authors:** Ivo Julião, Telma Costa, Lina Antunes, Paulo Almeida, Paulo Salamanca, Hirondina Borges, Lúcio L Santos

**Affiliations:** 1 GONCO Initiative - Associação de Investigação de Cuidados de Suporte de Oncologia, 4400 Vila Nova de Gaia, Portugal; 2 School of Public Health, The University of Sydney, Sydney, NSW 2050, Australia; 3 Hospital Central do Lubango, Lubango, Angola; 4 Hospital Batista de Sousa, Mindelo, Cabo Verde; 5 Scientific and Educational Directorate of the Cacuaco General Hospital, Luanda, Angola; 6 Oncology Department, Hospital Agostinho Neto, Plateau 112, Praia, Cabo Verde; 7 Experimental Pathology and Therapeutics Group, Portuguese Institute of Oncology and University of Fernando Pessoa, 4200 Porto, Portugal; 8 Surgical Oncology Department, Portuguese Institute of Oncology, 4200 Porto, Portugal; a https://orcid.org/0000-0003-0660-9215; b https://orcid.org/0000-0002-9625-1124; c https://orcid.org/0000-0002-3231-3162; d https://orcid.org/0000-0002-0521-5655

**Keywords:** Portuguese-speaking African countries, Angola, Cape Verde, global health, oncology services, cancer care, low- and middle-income countries, decentralised oncology, health systems strengthening

## Abstract

Cancer burden is expected to increase in the next decades, especially in low- and middle-income countries (LMICs). There, including in Portuguese-speaking African countries, cancer care remains fragile and highly centralised. Global ONCOlogy Initiative (GONCO) is a pragmatic initiative launched in Portugal to support the development of decentralised oncology services through short, targeted interventions. This paper presents its conceptual design and the first two pilot projects, implemented in Lubango (Angola) and Mindelo (Cape Verde).

GONCO follows a three-step model: digital planning, fieldwork and digital follow-up. In both sites, the program was co-developed with local teams and focused on establishing multidisciplinary tumour boards, improving service coordination and building capacity in clinical management, protocols and research. In Lubango, GONCO helped launch the oncology unit and research group. In Mindelo, it supported service restructure planning and the creation of a breast cancer working group. Remote collaboration sustained momentum after field visits. Challenges included hierarchical barriers, unreliable digital infrastructure and non-sustainable funding. Despite these, GONCO demonstrated that focused and adaptable models can catalyse oncology development in resource-constrained hospitals. GONCO provides a replicable, light-footprint model for early oncology development in LMICs.

## Introduction

The global burden of cancer incidence and mortality is rapidly growing and will soon be the leading cause of premature death worldwide [1, 2]. Projections estimate that by 2040, more than 28 million cancer cases will be diagnosed, representing a 47% increase compared to 2020 [1]. The increase in incidence and mortality will be more striking in low- and middle-income countries (LMICs), with 70% of all cancer deaths expected to occur in these regions [3]. This poses a significant threat to LMICs healthcare systems, already stretched thin [4].

The same happens in Portuguese-speaking African countries (PALOP). They form a collaborative group of African nations that includes Angola, Mozambique, Guinea-Bissau, Cape Verde and São Tomé and Príncipe. Despite their distinct identities, PALOPs have a long tradition of cooperation, which enabled the creation of effective transnational networks in oncology, such as the PALOP School of Oncology or the AORTIC-PALOP [5, 6]. These countries also face common challenges in cancer control: centralised and siloed services, fragile referral pathways, competing needs and priorities and lack of data to inform decision-making [5]. Over the past decades, they developed initiatives focusing mainly on primary prevention and screening, but without effective models for the management of advanced disease [6–8].

The Global ONCOlogy Initiative (GONCO) is a Portuguese alliance created to address some of these challenges. It is hosted by Associação de Investigação de Cuidados de Suporte em Oncologia, a Portuguese not-for-profit organisation, in collaboration with the PALOP School of Oncology. Its purpose is to work collaboratively towards the improvement of cancer care in LMICs, particularly in PALOPs, while contributing to the achievement of the 2030 Sustainable Development Goals [9]. It intends to decentralise cancer care by providing a rapid, context-adapted intervention to support the development of small oncology units in remote regions.

In this article, we present GONCO’s conceptual design for its two inaugural projects in Lubango (Angola) and Mindelo (Cape Verde). The authors also share their initial findings and lessons learned.

## Methods

GONCO is an innovative approach that focuses on co-developing the oncology framework within institutions and working with several collaborators for a comprehensive and complementary approach to all technical needs. This enables not only the development of the oncology department, but also the integration of all cancer-related services, serving as the foundation for effective cancer care delivery, while avoiding duplication and siloed implementation. It combines several pragmatic, experience- and literature-driven core principles of implementation [10–19], such as teaming up with motivated local teams, continuously adapting to local contexts, decentralising cancer care and focusing on critical structures like the multidisciplinary tumour board (MTB). They are distributed across five different categories: strategic vision and sustainability, local partnership and capacity building, contextual adaptation, innovation and digital tools and policy and advocacy (Table 1).

These principles are operationalised through a structured three-step model, described below.

*GONCO: a combined approach*

GONCO follows a three-step operational model:

Step 1: Digital planning

Step 2: Fieldwork

Step 3: Digital follow-up

Each project is implemented in cooperation with an in-country partner institution (ideally, a secondary or tertiary hospital) and tailored to its specific needs and priorities. Upon completion, a new project phase can be initiated with renewed objectives and procedures.

### Step 1: Digital planning

A local team is appointed by the partner institution to work directly with GONCO, starting with a series of virtual meetings. These sessions aim to explore the local context, define preliminary objectives and plan initial activities. They also promote team cohesion, while reducing the length and intensity of on-site activities.

### Step 2: Fieldwork

In this step, at least two GONCO members visit the partner institution from 3 to 12 weeks. Together with the local team, they start by performing a comprehensive setting evaluation, visiting facilities, interviewing relevant staff and engaging with leadership. Following this, the implementation of the planned activities starts.

### Step 3: Follow-up

This final step provides continued support after fieldwork. It consists of regular virtual meetings, with periodicity and duration to be defined by each team, for a defined period. They intend to promote sustainability and to co-design new projects.

### Selecting institutions

Partner institutions are public hospitals located away from the national cancer centre, that are either initiating cancer care or undergoing significant improvements, with the agreement and support of local and/or national authorities.

### Monitoring and evaluation (M&E)

The M&E strategy was embedded in the implementation approach. It was designed to address the feasibility and sustainability of the interventions rather than to measure clinical outcomes. The implementation outputs are under further analysis by the local institutions for future publication.

The key indicators monitored were:

- The establishment of an MTB and continuity of regular meetings;
- The delivery and attendance of oncology training sessions and evaluation of knowledge, attitudes and practices;
- The development and routine use of local clinical protocols and referral pathways;
- The initiation and conclusion of locally led clinical research activities;
- The persistence of key activities during the digital follow-up phase.

The evaluation used mixed methods (quantitative and qualitative) that consisted of direct field observation, documentation reviews (e.g., MTB meeting records), surveys and informal assessment interviews to collect feedback from institutional leaders and team members.

A new long-term assessment is planned for 2025 and will be published separately.

## Results

**Figure 1.**
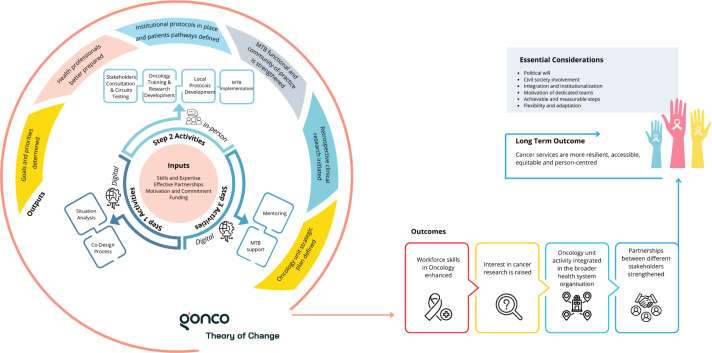
Theory of change of GONCO. Outlines the inputs, activities of the three steps, outputs, outcomes (intermediate and long-term) and other considerations for successful implementation. MTB, Multidisciplinary Tumour Board.

**Figure 2.**
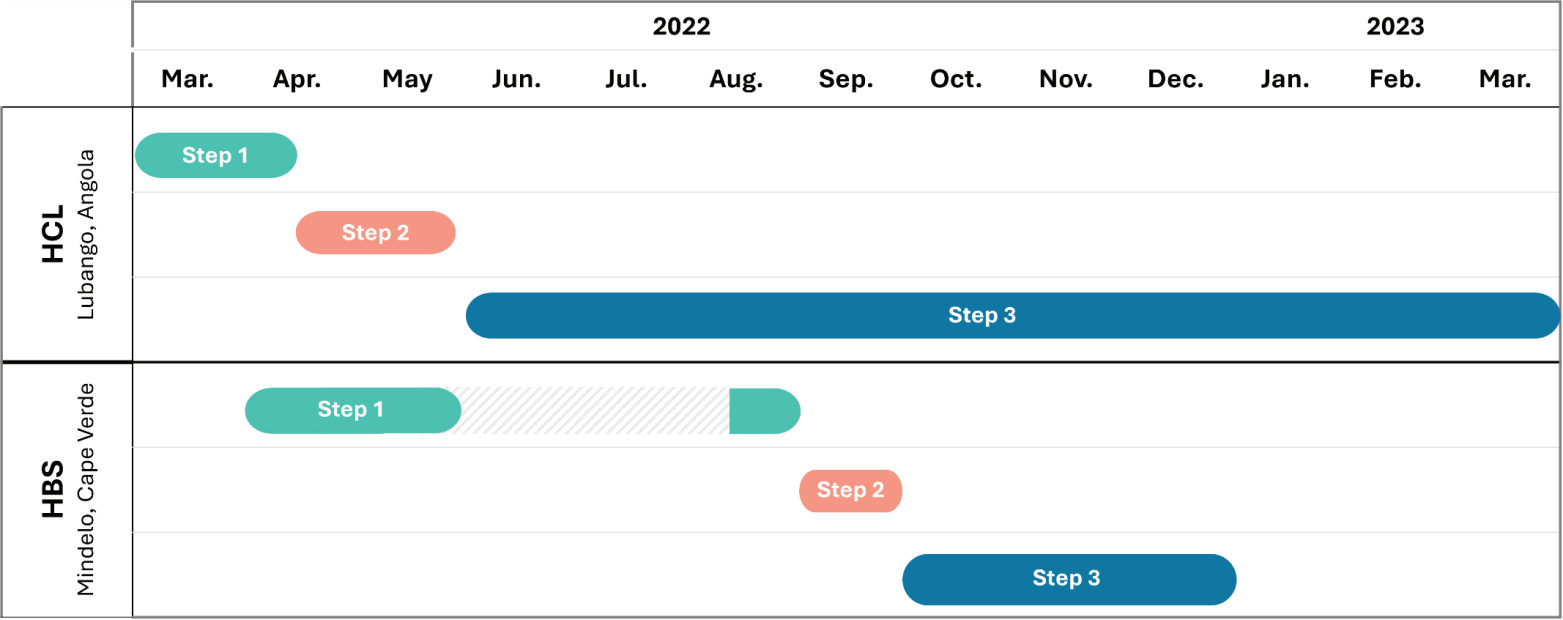
Timeline of GONCO pilot projects. Describes the complete timelines and duration of both projects and their steps. In HCL, Angola: Step 1 - digital planning (6 weeks); Step 2 – fieldwork (6 weeks); Step 3 – digital follow-up (10 months). In HBS, Cape Verde: Step 1 - digital planning (9 weeks); Step 2 – fieldwork (3 weeks); Step 3 – digital follow-up (3 months duration). HBS, Hospital Dr. Batista de Sousa. HCL, Hospital Central de Lubango.

During 2022 and 2023, GONCO completed its first two pilot projects in two different locations. The first was developed at Hospital Central de Lubango (HCL) in Lubango, Huíla province of Angola and the second at Hospital Dr Baptista de Sousa (HBS) in Mindelo, São Vicente Island, of Cape Verde. In these two countries, the development of decentralised oncology units is part of the national strategy to improve cancer care, in the pursuit of overcoming major inequalities between regions. The theory of change, with GONCO’s main activities, outputs and outcomes, is presented in Figure 1, and the timeline and duration of the different steps of both projects are in Figure 2.

## Lubango, Angola

### Setting and context

Angola is a large country in Central Africa, with an estimated population of 33,086,278 people in 2022 [20], and a cancer incidence of 24,607 new cases and 15,541 cancer-related deaths each year [21]. Angola is a lower middle-income country with only one public cancer center, the Instituto Angolano de Controlo do Cancer, located in the capital, Luanda, in the north of the country [22, 23].

In the southern Huíla province, HCL is responsible for the diagnosis and staging of most cancer patients within that region, serving directly a population of more than 2 million citizens [24]. Despite being equipped for diagnosis and staging, treatment options are mainly limited to surgery for early cases. Many patients need to be referred to Luanda for further treatment, causing a heavy financial and social burden for patients and families. Currently, HCL is building the first oncology unit outside Luanda. Concurrently, its Pathology Department is being strengthened and the regional population-based cancer registry started early in 2022.

### Step 1: Digital planning

The digital planning phase began in March 2022. GONCO team held a series of virtual meetings with the local team, hospital leadership and other stakeholders and jointly defined the following objectives:

- Develop the Oncology Unit as a central structure for cancer care within the institution, establishing links between other cancer-related services (pathology, general surgery, urology, gynecology and internal medicine);
- Improve the management of four of the most prevalent cancers: breast, cervix, prostate and skin cancer in people with albinism;
- Engage all relevant hospital departments in integrated cancer care delivery;
- Strengthen oncology education and training across professional categories (specialists, residents and nurses).

An overview of the findings on local resources for cancer care is available in Table 2.

### Step 2: Fieldwork

The fieldwork phase was conducted over a 5-week period, from April to May 2022. During that time, the oncology unit was inaugurated, although still without the infrastructure needed for chemotherapy treatments. GONCO supported the following developments:

- Definition of patient navigation strategies;
- Standardisation of diagnosis and staging protocols;
- Adaptation of clinical resources and guidelines across departments;
- Drafting of a short- to medium-term clinical and management strategy for the unit;
- Development of a hospital-wide palliative care implementation plan.

Training was conducted in parallel. A 28-hour foundational course in oncology was delivered to 22 participants, with support from the PALOP School of Oncology. Post-course knowledge assessment indicated improvement compared to baseline. Other activities included workshops, journal clubs, group discussions and case-based training with different departments.

GONCO also facilitated the creation of a research team with members from several services of the hospital. After initial training, the team developed multiple cross-sectional study protocols, addressing the main target cancers, as well as other locally relevant conditions, such as brain metastasis. The protocols were approved by the local ethics committee, and data collection is ongoing. The group also prepared a development plan in collaboration with the hospital’s research department.

A weekly MTB was established for all cancer patients admitted to the hospital. National engagement was secured by the participation of a remote medical oncologist, alongside a local surgical oncologist. It is now institutionalised and actively managed by the local team.

### Step 3: Digital follow-up

A structured virtual follow-up phase was maintained for 10 months, with weekly coordination sessions between GONCO and the local team. This included discussion of clinical cases, journal club sessions, training activities, planning for unit development and research. At the end of this period, the teams held a meeting with the hospital board to review the oncology unit’s early achievements.

During this phase, GONCO continued participating in the MTB meetings, which served as a central platform for coordination and shared decision-making.

## Mindelo, Cape Verde

### Setting and context

Cape Verde is a West African LMIC composed of ten islands located in the Atlantic Ocean, with a population estimated at 491,233 people [25]. In 2022, 435 new cancer cases were diagnosed, and cancer was the second leading cause of death in Cape Verde in 2017 [26, 27]. There is only one oncology unit in the country, in Hospital Agostinho Neto (HAN), located in the capital city, Praia, in Santiago Island, to which cancer patients from other islands need to be referred. Moreover, patients requiring radiotherapy or more complex treatment approaches have to be transferred to Portugal, as part of a bilateral agreement, which poses a major logistical and financial challenge [27]. At HBS, in Mindelo, São Vicente Island, oncology is developing on multiple fronts. An international surgical fellowship program provided the HBS with the first surgeon trained in surgical oncology [28], and a population-based cancer registry started in early 2022 [28]. There is also a small, long-standing team dedicated to the management of non-surgical cancer patients, although they lack the capacity to perform chemotherapy. These services are intended to cover more than 95,000 inhabitants [25]. A new Ambulatory Centre is currently under construction, with plans for an area dedicated to comprehensive cancer care, including a pharmacy for chemotherapy compounding and a day hospital for systemic cancer treatments.

### Step 1: Digital planning

GONCO held a series of virtual meetings with the Clinical Director of HBS, between April and June 2022. Due to several contextual challenges, such as a lack of junior staff, limited availability and unfamiliarity with co-development strategies, step 1 took place with Hospital’s leadership and not the local team. As a result, most co-development activities were deferred to the early days of step 2.

Four priority cancer types were identified – breast, cervix, gastric and esophagus – and three main objectives were defined:

- To conduct a comprehensive assessment of oncology services
- To develop a short-to-medium-term strategy for the unit
- To plan the oncology unit transition to the new Ambulatory Center.

### Step 2: Fieldwork

The fieldwork was conducted over 3 weeks in September 2022. Despite the challenges in engaging the pre-assigned team, the main objectives were achieved. An informal team of motivated professionals was assembled to give support to the implementation.

A comprehensive assessment of the oncology unit and related departments was carried out through direct observation, staff interviews and meetings with the National Cancer Program and civil society organisations. Table 2 summarises the assessment of resources done during steps 1 and 2.

GONCO developed a restructuring proposal focused on service integration, workforce planning, the formalisation of the MTB and the integration of oncology into the Hospital’s broader clinical strategy. The proposal was approved by the Hospital board.

A weekly MTB was established, with remote support from HAN and coordinated through a mobile messaging group. A breast cancer working group was also created, involving professionals from surgery, gynaecology and medical imaging. This group developed diagnostic and treatment strategies to streamline breast cancer management.

Most of the educational activities were delivered through the MTB sessions.

### Step 3: Digital follow-up

For 3 months following step 2, GONCO maintained digital support to assist with the implementation of the restructuring plan and to consolidate the newly created MTB. Weekly virtual meetings were held with selected local stakeholders to address challenges, provide technical input and maintain engagement.

## Discussion

GONCO aims to provide simple, sustainable and targeted solutions to support the co-development of decentralised oncology units and ultimately contribute to improving outcomes for cancer patients in LMICs. While this report has demonstrated the feasibility of the model and its tangible outputs and early outcomes (Figure 1), it is equally important to discuss the challenges encountered, key lessons learned and practical implications for future implementation in similar settings. To the best of our knowledge, GONCO is the first and only program currently dedicated to the development of small oncology units in decentralised institutions in the PALOPs.

### Implementation outcomes in Lubango and Mindelo

GONCO’s three-step model – digital planning, fieldwork and digital follow-up – proved feasible in two distinct contexts. Its success was driven by core principles, primarily flexibility, adaptability and sustainability aiming to develop capacity without fostering dependency. This strategy aligns with initiatives like the City Cancer Challenge (C/CAN) [29], promoting context-driven, locally led solutions.

In Lubango, GONCO co-developed an oncology unit, launched a weekly MTB, provided training across departments and supported the formation of a research team. This shows the impact of focused and co-developed efforts in leveraging existing resources in low-resource settings. Like the AMPATH-oncology model in Kenya [30], GONCO combined mentoring, training and service integration, but with a lighter operational footprint.

In Mindelo, GONCO showed adaptability in a fragmented institutional environment. Despite the difficulties of engaging with the pre-established team, key outputs were achieved: a restructuring proposal for the oncology unit, the creation of a breast cancer working group and the establishment of a functioning weekly MTB with support from HAN. GONCO’s detailed context evaluation echoed World Health Organisation – International Atomic Energy Agency (WHO–IAEA) imPACT reviews [31], but went further by translating systemic gaps into actionable, site-specific plans.

### Strengths and strategic advantages of GONCO

GONCO was designed to be time-bound, low-cost and with a light operational footprint. This contrasts with broader initiatives such as C/CAN [29] or IAEA’s imPACT reviews [31], that require long-term engagements or large technical missions. GONCO delivers short and focused fieldwork supported by virtual coordination. This reduces the logistical burden on host-institutions, avoids long-term dependency on external actors, reduces costs and facilitates implementation in resource-limited settings.

The use of digital tools was key to supporting both planning and follow-up. Remote participation in MTB meetings, team sessions and messaging groups extended the project’s reach and helped reinforce its continuity without requiring permanent on-site presence.

GONCO’s adaptability was tested in Cape Verde, where a fragmented staff engagement made it difficult to work with the pre-established local team. The project moved forward by mobilising a different group of motivated professionals. This flexibility in stakeholder engagement proved essential for attaining the outcomes. This illustrates how core values can be pragmatically applied to enable implementation in real-world LMIC settings.

MTBs emerged as one of the most transformative outputs of the GONCO projects. In both settings, this new structure became central to case management, cross-department collaboration and professional cohesion. They also served as training platforms and institutional dialogue. Their sustained use demonstrates their relevance and acceptability and shows the potential to anchor the development of cancer services in fragmented or resource-limited settings.

Decentralisation is a critical dimension of GONCO’s approach, enabling oncology development in secondary urban centres like Lubango and Mindelo. In LMIC’s, where access is often centralised, this strategy helps reduce inequality and treatment delays [32]. The WHO Global Initiative for Childhood Cancer [33] recognised the importance of decentralising and other authors have underscored the need to build capacity at subnational levels to reduce disparities [34]. Instead of just replicating tertiary centres, GONCO focused on developing key capacities such as the MTB, diagnosis, referral pathways, navigation, palliative care and training, within districts and provincial hospitals. This focused model reduces pressure on referral hubs and improves timely access to care.

GONCO operated within a broader collaborative network where different organisations addressed specific technical components (pathology, infrastructure and equipment) while GONCO focused on the essential oncology framework – the organisational glue that enables all components to function together. Ongoing clinical mentorship through MTBs, return visits and regular communication groups with national and international specialists provided sustained clinical support beyond the initial implementation period. These strategies will be the focus of future publications.

Finally, GONCO fills an important implementation gap between large-scale initiatives and the operational needs of district or provincial hospitals in LMICs. It brings a realistic and scalable entry point for initiating cancer services where oncology is still emerging as a structured field. Its approach avoids high logistical burdens or long-term external commitments, while generating tangible, time-bound results. This presents GONCO as an adaptable tool to catalyse the development of cancer services in resource-constrained settings, especially where national cancer strategies are not present or developed at the subnational level.

### Challenges and lessons learned

The GONCO experience showed several challenges for implementation. In Mindelo, staff availability, hierarchical dynamics and unfamiliarity with co-development approaches hindered early engagement. This underscores the importance of early engagement, mapping institutional dynamics, addressing expectations and building mutual ownership from the onset. The purpose-driven nature of GONCO can also become a challenge, demanding a strong local leadership to ensure continuity after fieldwork. In both projects, connectivity issues affected remote coordination and training, highlighting the importance of a timely assessment of the digital infrastructure and to plan support accordingly.

Finally, GONCO’s reliance on restricted philanthropic funding limited its scope and flexibility. Although the model never intended to establish full oncology units in a single project, its sustainability depends on securing follow-up support for sequential projects or the integration into broader national projects. This can raise the opportunity to explore new financial models that combine multiple-sourced investments, in conjunction with local commitment.

All these challenges reflect common realities in LMIC implementation projects and reinforce the need for adaptable planning, sustained engagement and long-term vision when initiating decentralised cancer care.

### Implications for replication

The results and challenges of this project offer practical lessons for similar interventions:

- Map stakeholders early to understand institutional barriers, align expectations and ensure shared ownership from the outset.
- Engage motivated individuals, even if outside formal hierarchies – flexibility in team composition proved more effective than rigid structures.
- Anchor work around the MTB, which served as a bridge across departments, improved clinical coordination and fostered connections with national cancer centres.
- Use digital tools to support planning and follow-up – this helped maintain momentum and institutional engagement after field visits.
- Assess digital infrastructure beforehand – reliable internet and mobile connectivity were critical for sustaining remote coordination and clinical discussion.

### Study limitations

This paper describes the development of an implementation model and its application in two projects, but does not assess clinical impact or long-term sustainability. Although following the initiative’s design, this limits the interpretation of its broader relevance. Furthermore, the model was tested in only two settings, that despite their diversity, may reduce generalisability. Lastly, the analysis was conducted by members of the implementation team, introducing potential bias.

### Future directions

GONCO will continue to support Lubango and Mindelo to consolidate current developments through follow-up assessments, focused on sustainability and outcomes of key structures such as MTB, research teams, protocols and patient navigation. As more data becomes available, measurable outcomes will be reported.

GONCO also aims to expand to new institutions, applying its core principles and building on the lessons learned. To enhance sustainability, future work may be embedded into broader initiatives or be delivered as successive, goal-driven projects that contribute to the long-term development of oncology in LMICs.

## Conclusion

The development of decentralised oncology services in low-resource settings remains a major challenge, including in PALOPs. GONCO offers a practical model through a coordinated, context-adapted and time-bound approach. The two pilot projects proved feasible and responsive to local needs. While long-term outcomes are still under evaluation, GONCO demonstrates how focused and adaptable interventions can catalyse cancer care development where it is more needed, offering a path that can be echoed in similar settings.

## List of abbreviations

AICSO, Portuguese Association for Supportive Oncology Research (Associação de Investigação de Cuidados de Suporte em Oncologia); C/CAN, City Cancer Challenge; CSO, Civil Society Organisation; CT, Computed Tomography; GONCO, Global ONCOlogy Initiative; HAN, Hospital Agostinho Neto; HBS, Hospital Dr. Batista de Sousa; HCL, Hospital Central do Lubango; IHQ, Immunohistochemistry; LMIC(s), Low- and Middle-Income Country(ies); M&E, Monitoring and Evaluation; MRI, Magnetic Resonance Imaging; MTB, Multidisciplinary Tumour Board; PALOP, Portuguese-Speaking African Countries (Países Africanos de Língua Oficial Portuguesa); US, Ultrasound; WHO–IAEA, World Health Organisation – International Atomic Energy Agency.

## Conflicts of interest

The authors declare that they have no competing interests.

## Figures and Tables

**Table 1.** GONCO core principles of work to support the co-development of decentralised oncology units in LMICs.

Category	Core principles
Strategic vision and sustainability	Focuses exclusively on cancer care and oncology services. Pushes to decentralise cancer care by supporting the development of decentralised units. Starts by developing critical structures/actions: oncology department, palliative care, MTB and research team. Doesn’t develop projects that are financially dependent on GONCO. Develops on top of national/regional plans, strategies, ideas and expectations.
Local partnership and capacity building	Works with a local team of highly motivated people and/or champions with whom it co-designs each project, making sure to provide training opportunities, transformative leadership and supportive supervision. Studies power structures, expectations and emotional dynamics within institutions or teams.
Contextual adaptation	Actively and continuously adapts to cultural, social, spiritual, political, educational and resource settings. Embraces reality in the field and uses it in the benefit of the project. Considers that small and tangible steps are sometimes better than big, unstructured undertakings.
Innovation and digital tools	Explores the use of digital solutions.
Policy and advocacy	Advocates for cancer in all policies. Sees in each stakeholder a potential cancer care advocate

**Table 2.** Summary of cancer care services availability in Lubango, Angola, and in Mindelo, Cape Verde, per GONCO assessment in 2022.

	HCL - Lubango, Angola	HBS - Mindelo, Cape Verde
Local team assigned to work with GONCO	Team Leader: Clinical Director of HCL Team Members: three oncology nurses, two general surgery residents, one coordinator of the regional cancer registry	Team Leader: Clinical Director of HBS Team Members: one oncology nurse, two general surgeons, one coordinator of the regional cancer registry
Type of health facility	Tertiary level hospital	Tertiary level hospital
Population served	3,282,968	75,845 people
Estimated number of cancer patients evaluated in the hospital/year	480	100
Organised population-based cancer screening programs	No - Opportunistic screening for cervical (cytology) and breast cancer (mammography)	Yes – cervical (cytology) and breast cancer (mammography)
Cancer registry	Regional population-based registry - started in 2022 (data contributed to Globocan 2022 (version 1.1) - 08.02.2024[21])	Regional population-based registry started in January 2022
Research unit / department	No	No
Oncology unit open and functional	No (planned to open in 2022)	Yes (since 2008)
Diagnosis and staging (routinely available)	Histopathology, no IHQ available X-ray, mammogram, US, CT scan (contrast use limited), MRI (limited)	Histopathology, no IHQ available X-ray, mammogram, US, CT scan (contrast use limited), no MRI available
Cancer management modalities (routinely available)	Surgery (surgical oncology available) No systemic treatment No radiotherapy No palliative care	Surgery (surgical oncology limited) Hormonotherapy and some oral chemotherapy, upon first evaluation and prescription in the Oncology unit of HAN, Praia No radiotherapy No palliative care
Organised MTB	No	No
CSO involved in cancer advocacy	Yes – Bolsa Solidária, based in Lubango	Yes – Cape Verdean league against cancer, based in Mindelo

CSO – civil society organisation; CT – computed tomography; HAN – Hospital Agostinho Neto; HBS – Hospital Dr. Batista de Sousa; HCL – Hospital Central do Lubango; IHQ – immunohistochemistry; MRI – magnetic resonance imaging; MTB – multidisciplinary tumour board; US – ultrasound
